# A low-power ankle-foot prosthesis for push-off enhancement

**DOI:** 10.1017/wtc.2023.13

**Published:** 2023-06-15

**Authors:** Alessandro Mazzarini, Matteo Fantozzi, Vito Papapicco, Ilaria Fagioli, Francesco Lanotte, Andrea Baldoni, Filippo Dell’Agnello, Paolo Ferrara, Tommaso Ciapetti, Raffaele Molino Lova, Emanuele Gruppioni, Emilio Trigili, Simona Crea, Nicola Vitiello

**Affiliations:** 1 The BioRobotics Institute, Scuola Superiore Sant’Anna, Pisa, Italy; 2 Department of Excellence in Robotics & AI, Scuola Superiore Sant’Anna, Pisa, Italy; 3 IUVO S.r.l., Pisa, Italy; 4 Department of Physical Medicine and Rehabilitation, Northwestern University, Chicago, IL, USA; 5 Max Nader Laboratory for Rehabilitation Technologies and Outcomes Research, Shirley Ryan AbilityLab, Chicago, IL, USA; 6 Institute of Recovery and Care of Scientific Character (IRCCS), Fondazione Don Carlo Gnocchi Florence, Firenze, Italy; 7 Centro Protesi Inail di Vigorso di Budrio, Bologna, Italy

**Keywords:** Biomechanics, control, design, mechatronics, prosthetics

## Abstract

Passive ankle-foot prostheses are light-weighted and reliable, but they cannot generate net positive power, which is essential in restoring the natural gait pattern of amputees. Recent robotic prostheses addressed the problem by actively controlling the storage and release of energy generated during the stance phase through the mechanical deformation of elastic elements housed in the device. This study proposes an innovative low-power active prosthetic module that fits on off-the-shelf passive ankle-foot energy-storage-and-release (ESAR) prostheses. The module is placed parallel to the ESAR foot, actively augmenting the energy stored in the foot and controlling the energy return for an enhanced push-off. The parallel elastic actuation takes advantage of the amputee’s natural loading action on the foot’s elastic structure, retaining its deformation. The actuation unit is designed to additionally deform the foot and command the return of the total stored energy. The control strategy of the prosthesis adapts to changes in the user’s cadence and loading conditions to return the energy at a desired stride phase. An early verification on two transtibial amputees during treadmill walking showed that the proposed mechanism could increase the subjects’ dorsiflexion peak of 15.2% and 41.6% for subjects 1 and 2, respectively, and the cadence of about 2%. Moreover, an increase of 26% and 45% was observed in the energy return for subjects 1 and 2, respectively.

## 1. Introduction

**Figure 1.**
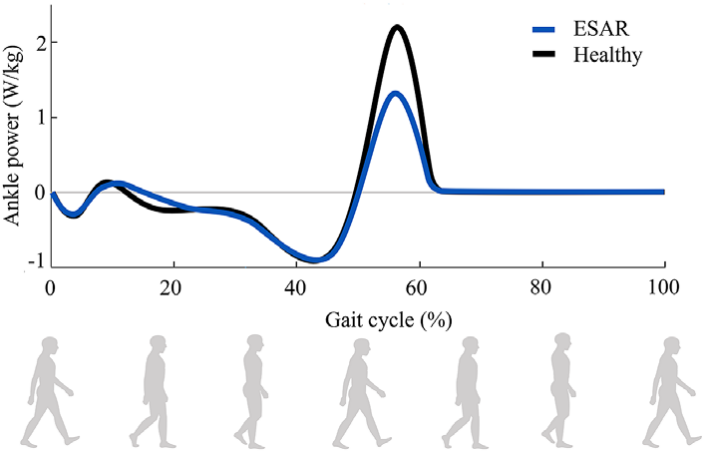
Joint power profiles of a standard ESAR foot and a healthy ankle in a complete gait cycle. The presented data are adapted from the study of Ferris et al. (2012).

Every year, tens of thousands of people undergo amputation in Europe and USA (Ziegler-Graham et al., 2008; Narres et al., 2017). In particular, lower-limb loss causes a disruptive change in the quality of life of amputees, reducing mobility in most activities of daily living (ADLs) (Whittle, 2014). Below-knee (i.e., transtibial) amputees have a slower speed and a metabolic consumption up to 20% higher than healthy individuals during Ground Level Walking (GLW) (Waters and Mulroy, 1999). Moreover, the energy lacking from the missing ankle joint is usually balanced with compensatory movements, which lead to abnormal gait patterns and asymmetry (Waters and Mulroy, 1999; Ferris et al., 2012; Windrich et al., 2016). Despite the ability of energy storing and return (ESAR) prostheses to enhance push-off, passive devices cannot generate net-positive ankle work or change their mechanical properties to match the angle-torque requirements of different gait phases or motor tasks (Au et al., 2009). Indeed, the power profile generated by an off-the-shelf ESAR foot during GLW closely matches the one of a healthy subject (Figure 1) except for the push-off phase, in which it is 45% lower than the positive power generated by a healthy ankle (Ferris et al., 2012).

Active ankle-foot prostheses restore more natural and efficient ambulation by directly driving the joint (Au et al., 2009; Gabert et al., 2020) or by mimicking the effects of the missing calf muscles (Hitt et al., 2010). However, the efficacy of powered devices is limited by the added distal mass and encumbrance due to large motors and power sources (Browning et al., 2007; Lenzi et al., 2019). Therefore, semi-active prostheses try to overcome the limitations of weight and encumbrance by integrating smaller actuators and lighter assemblies.

Among commercial products and prototypes developed over the past years, most semi-active prostheses improve the stability and comfort of amputees across different tasks by changing the foot’s mechanical properties (e.g., stiffness) during non-weight-bearing gait phases (Shepherd and Rouse, 2017; Glanzer and Adamczyk, 2018; Lenzi et al., 2019). Usually, the ankle stiffness is modulated to match the torque-angle relationship of different locomotion modes. Alternatively, the ankle angle may be modulated to (i) enhance foot clearance (Proprio Foot, Ossür®, Reykjavik, Iceland) (Meridium, Ottobock®, Duderstadt, Germany), (ii) adapt to different terrains and shoes (Lenzi et al., 2017; Lenzi et al., 2019), and (iii) align to inclines (Elan, Blatchford®, Basingstoke, United Kingdom). A third approach is to power specific gait phases. In fact, some of the semi-active devices can operate during the stance phase (Collins and Kuo, 2010; Cherelle et al., 2017) to harvest energy in additional passive elastic elements and return this energy at push-off, restoring a more natural and efficient gait (Caputo and Collins, 2014). In the literature, few devices have been developed following this design approach. The energy recycling foot uses a clutched system to engage a parallel elastic element at the heel strike (Collins and Kuo, 2010). The energy developed during foot contact is retained in the spring until push-off when the elastic energy is transferred to the fore part of the prosthesis for forward propulsion. Instead, the AMP-Foot 3 uses an actuator to load a spring during stance and return it at push-off (Cherelle et al., 2017). Moreover, the compression of two additional parallel springs by the user weight provides stability and controlled dorsiflexion during early and mid-stance. When the push-off occurs, all the energy stored in the elastic element and the parallel springs is delivered to the ankle joint, producing the total power required for forward propulsion. This design approach has been proven to reduce the net metabolic expenditure of walking (Collins and Kuo, 2005) at the expense of added mechanical complexity and increased device energy consumption. Mechanical complexity may be reduced if a passive ESAR foot is used as structural frame for the actuation, while taking advantage of the ESAR elastic properties.

In this study, we present the Wearable Robotics Lab TransTibial Prosthesis (WRL TTP), a low-power device designed to improve the energy-storing performance of a passive ESAR foot during locomotion. The WRL TTP fundamentally differs from previous semi-active transtibial prostheses as it is equipped with a parallel actuator to increase the deformation of the foot, and thus, to harvest energy to be returned at a tunable stride phase of the gait cycle. Hereby, we present the concept verification of the device on two transtibial amputees.

This article is organized as follows: Section 2 presents the design of the low-power transtibial prosthesis. Section 3 reports the methodology and results of the experimentation with amputees. Section 4 discusses the results and Section 5 draws the conclusions.

## 2. WRL TTP platform

**Figure 2.**
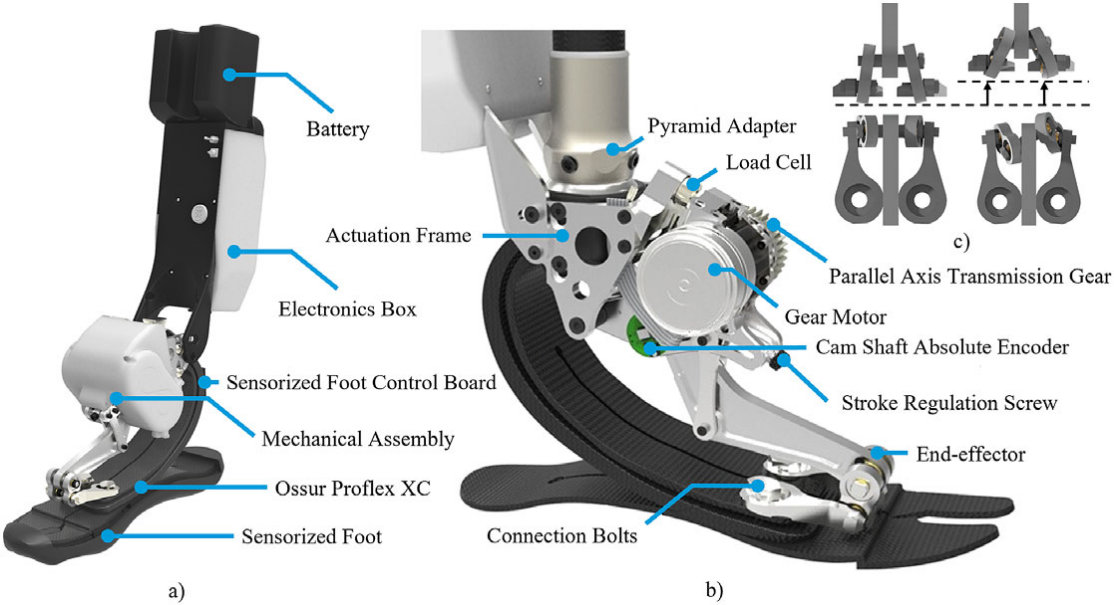
(a) Overview of the WRL TTP and its main components. (b) Rendering of the lateral view of the mechanical assembly of the WRL TTP without the cover and the sensorized foot. (c) Front and top view of the differential mechanism connecting the end-effector with the ESAR forefoot. The figures on the left show the differential mechanism in tension, while the ones to the right show the safe self-folded configuration.

The WRL TTP is a stand-alone ankle-foot prosthesis comprising an actuation unit embedded in parallel to a customized ESAR foot (Pro-Flex® XC C5 S27, Össur). The whole sensory apparatus, electronics, and battery are onboard, housed in a 3D-printed box on the back of the shank to reduce the encumbrance and the inertial effects due to the added mass on the foot. The electronics box can be adjusted along the sagittal plane thanks to a screw mechanism that couples the box and the actuation frame. This permits to accommodate wider amputee sockets and makes the prosthesis adaptable to different end-users (Figure 2(a)).

### 2.1. Actuation unit and sensing

**Figure 3.**
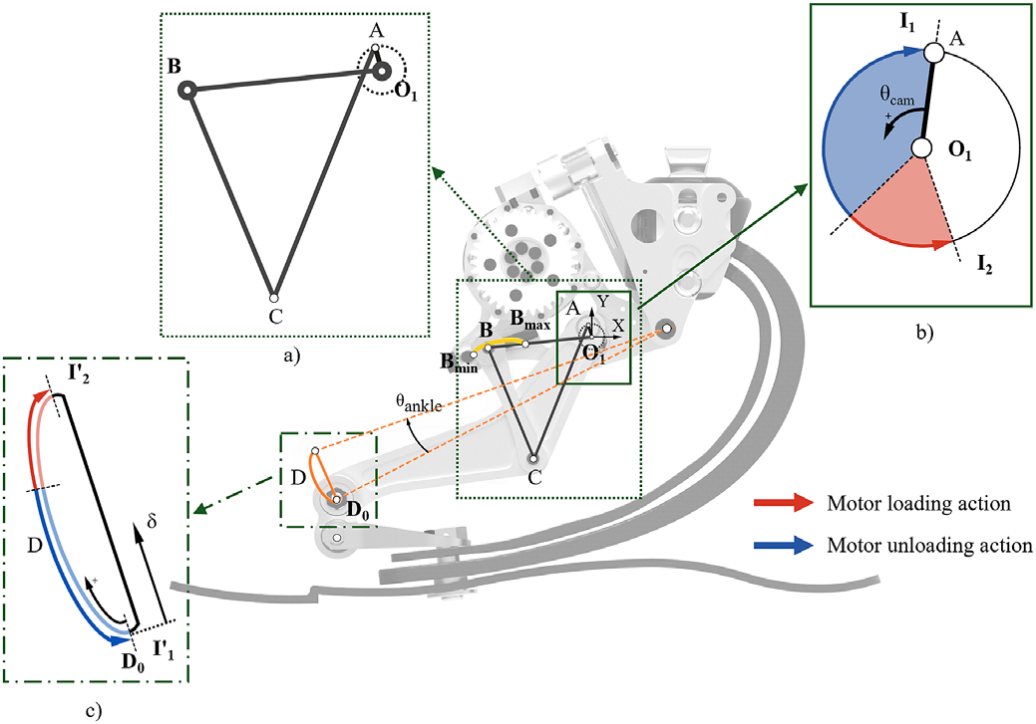
Mechanical assembly of the WRL TTP. (a) Segments of the four-bar linkage O_1_-A-C-B. (b) Camshaft trajectory with the two instability points of the mechanism I1 and I2. (c) End-effector trajectory. Bold letters correspond to fixed points.

The low-power actuation unit includes a compact 30 W brushless motor (EC45 flat, Maxon Motor®) equipped with an incremental encoder (2048 cpr, MILE) and followed by a 30:1 Harmonic Drive® (HFUC-11-2A-30). An overrunning clutch has been embedded in the reducer output shaft to allow free rotation in only one direction. A 1:1 nylon gear coupling transfers the motion from the motor shaft to the camshaft (link 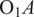 in Figure 3), delivering up to 4.5 Nm peak torque to the camshaft neglecting the efficiency losses introduced by the gears. The angular position of the camshaft 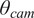 is measured by a 13-bit absolute encoder (RMB20 OnAxis™, RLS-Renishaw®). The actuator is interfaced with the forefoot and the pyramid adapter’s housing. A monoaxial load cell assembly in the upper constraint (8417-6001, Burster®) measures the force exchange in the sagittal plane between the leaf spring of the ESAR foot and the actuator (Figure 2(b)). The load transmitted to the load cell is reduced by a factor of 1.93. An under-constrained differential mechanism connecting the actuation unit to the ESAR foot (Figure 2(c)) ensures the prosthesis’ safety and essential mobility functions when the motor is turned off. The differential system is designed to (i) compensate for the horizontal misalignment between the motion of the end-effector and the shape of the forefoot when the structure of the ESAR foot is deformed, (ii) to even the interaction forces between the two sides of the bottom plate of the prosthetic foot to improve lateral stability, and thanks to the self-folding feature of the differential mechanism (iii) to avoid overloading the actuator end-effector when compression forces are applied during stance.

The WRL TTP can monitor the orientation of the limb segments using three Inertial Measurement Units (IMUs). One IMU is embedded in the main electronic board, monitoring the orientation of the shank (iNemo, LSM9DS1, STMicroelectronics), and the other two are wired and placed on the rear of the foot and the thigh of the user (MPU9250, TDK/InvenSense Inc.). The bottom plate of the foot is equipped with 16 wired pressure-sensitive elements based on optoelectronic technology (Fiumalbi et al., 2022), embedded in scalable sets of PCB matrices. The placement of the sensors follows the most stressed plantar areas, namely the heel and forefoot regions, to improve the detection of gait events such as foot contact and foot off. Threshold-based algorithms use the information from the pressure-sensitive elements to provide online estimates of the center of pressure ( 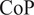 ) progression and the vertical ground reaction force ( 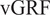 ). Detailed information can be found in Fiumalbi et al. (2022). Signals from pressure-sensitive elements are collected by the sensorized foot control board that interfaces with the main electronic board. The WRL TTP system weighs 2745 g: 665 g for the commercial ESAR prosthesis (foot cosmesis included), 630 g for the actuation unit, 550 g for the electronics and the sensory system, 500 g for the carter and the covers, and 400 g for the battery.

### 2.2. Parallel elastic actuation principle

In this implementation, the parallel elastic actuation paradigm (Fantozzi et al., 2018) allows both the user and the motor to compress the elastic structure of the passive foot. Hence, whenever the user loads the prosthesis, the leaf spring structure of the foot is bent. The actuator is designed to further compress the ESAR foot to increase its load and dorsiflexion, augmenting its energy storage capability. The elastic energy stored in the ESAR foot is retained by a four-bar linkage, defined by the points 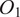 - 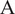 - 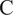 - 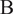 (Figure 3(a)). The base of the four-bar linkage is the link 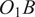 , fixed to the frame. By moving the link 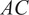 , the linkage provides a cyclic approximate linear motion of the end-effector 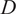 (Figure 3(c)), connected to the forefoot portion of the prosthesis, leading its vertical displacement along a complete cycle of the camshaft (Hoeckens linkage (Lu et al., 2014)). The linkage shows two instability points, 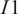 and 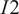 (Figure 3(b)), which divide the motion of the end effector 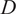 of the four-bar mechanism into two regions: from 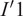 to 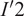 (corresponding to the points 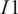 and 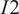 in the camshaft trajectory) the mechanism stores and retains the energy, whereas from 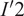 to 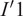 the energy is rapidly returned. This behaviour is ensured by the overrunning clutch positioned in the motor shaft. The camshaft angle ( 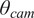 ) is set to 0 when the camshaft coincides with the instability point 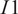 . The instability point 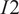 is reached when the camshaft angle is between 200 and 215 deg. Whenever the instability point 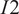 of the linkage is crossed (*flipover* event), the overrunning clutch allows the mechanism to move freely up to the instability point 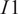 , thus enabling the release of the stored energy. Alternatively, if 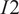 is not reached, the motor unloads the structure by bringing back the linkage at a controlled velocity up to the instability point 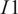 (blue arrow in Figure 3(b)). The linkage is brought back by driving the motor at negative velocities (motor shaft rotating counterclockwise and camshaft in the opposite direction).

A kinematic model was developed to estimate (i) the dorsiflexion angle ( 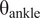 in Figure 3) and (ii) the displacement 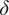 of the end-effector 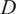 from its lowest position 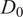 . These variables are used as input to a kinetic model to provide an estimate of the energy stored in the prosthesis. Additional information can be found in the Supplementary Material.

**Figure 4.**
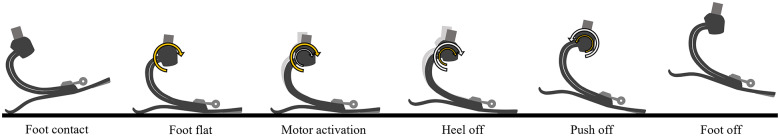
The assistive actuation concept is shown during stance. The arrows indicate the inversion of torque during the energy release at push-off. The yellow arrows are relative to the amputee’s loading action, while the light grey arrows are relative to the actuation’s loading action.

For any given force applied at the end-effector 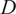 , the resulting torque magnitude and direction at the camshaft 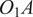 depends on the four-bar linkage geometrical configuration provided by the camshaft angle. In particular, the torque direction reverses at every flipover (Figure 4). The motor’s active deformation is meant to work in synergy with the loading action exerted by the user. Moreover, if the motor is driven at constant maximum velocity, the amount of deformation that the actuator can provide to the elastic structure of the foot depends on the time interval in which the motor can effectively engage the clutch to inject energy into the foot. This time window spans between the foot-flat and the push-off phases (loading phase), lasting about 40% of the stride phase. During this time window, the motor injects into the system the energy required to move the camshaft over 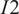 , starting from the position in which the camshaft is brought by the sole loading action of the user (red arrow in Figure 3(b)).

Since the actuator is meant to be engaged only in a small time window of the gait cycle, the motor shaft rotational velocity was the main specification that has been considered in the choice of the motor and the reducer. Thanks to the free motion of the overrunning clutch during the energy return phase at push-off, it is possible to avoid high-velocity requirements of the motor. The energy return is in fact unhindered from the actuator action due to the decoupling between the clutch and the motor shaft. Therefore, the maximum rotational velocity of the motor is a constraint only during the loading phase. Indeed, the motor must rotate the camshaft 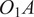 of about 205 degrees in 40% of the gait cycle duration ( 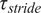 ), to allow the end-effector 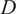 to span the range from 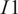 to 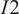 . By considering a 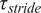 of one second, the velocity limit for the actuator in the loading phase was computed as:
(1)

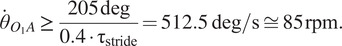

The system is designed to comply with different users and stiffnesses of the embedded ESAR foot. Notably, the geometry of the linkage can be manually regulated to adapt to the deformation profiles: the position of point 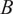 of the linkage can be adjusted between 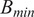 and 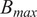 along a circular arc guide (yellow arc in Figure 3) by a screw mechanism, hence changing the stroke of the 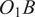 link. The implemented regulation (hereafter called *stroke regulation*) modifies the maximum vertical displacement of the end-effector trajectory while keeping approximatively at a fixed position the lowest point ( 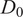 ), similarly to how a variable-stroke engine operates.

The actuation assembly allows the substitution of the camshaft gear to reduce the length 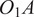 and therefore to shift the position of 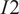 . In this case, the modified geometry of the transmission reduces the maximum vertical displacement of the end-effector, and it can be used to adapt the actuation unit to reduced deformation profiles, as in the case of high-stiffness ESAR feet or lightweight subjects (Sawers and Hahn, 2011). Hence, the presented system shows the possibility to be conveniently modified since the geometry of the actuator can be changed functionally to the available mechanical power and stiffness profile of the foot.

### 2.3. Electronics and control architecture

The WRL TTP is controlled by a real-time National Instruments SbRIO-9651 System on Module (SOM), equipped with a real-time processor Xilinx Zynq-7020 with dedicated FPGA. An IEEE 802.11 connection allows remote monitoring and control parameters setting via a graphical user interface (GUI). The prosthesis is powered by a 24 V battery pack (a series of 6 cells of Panasonic NCR18650PF) with an integrated Battery Management System that can guarantee more than two hours of continuous operation. The motor is driven by an Elmo Gold Twitter, with a limited current range equal to [−2 A, 2 A]. Its current supply can be interrupted by acting on an external emergency button in case of adverse events.

**Figure 5.**
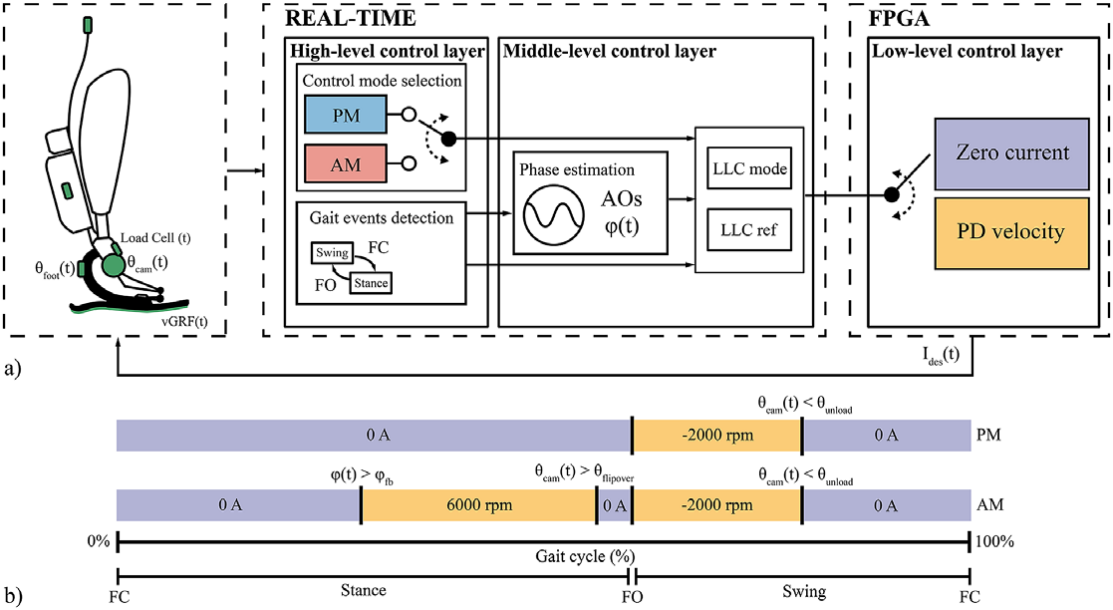
(a) Layered control architecture of the WRL TTP. The control mode selection in the High-Level layer is performed via manual selection on the remote GUI. (b) Graphical representation of the mode-specific assistive strategy in the middle-level layer. The violet parts indicate a low-level control in current (desired output in A). The yellow parts indicate a low-level control in velocity (desired output in motor rounds per minute).

The control architecture of the prosthesis is developed in three main layers (Figure 5), following the generalized framework developed in most current wearable robotic devices (Tucker et al., 2015). The high-level layer and middle-level layer run on the SOM real-time processor at 100 Hz, while the low-level layer runs at 1 kHz on the SOM FPGA.

1. *High-level control layer:* The high-level controller uses the GUI inputs on a remote laptop to select the control mode of the WRL TTP and to set control parameters and segmentation thresholds. Moreover, it is responsible for detecting gait events – Foot Contact (FC) and Foot Off (FO). The FC and FO events can be detected using either a threshold on the 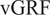 estimated from the sensorized foot or a rule-based algorithm on signals from the foot IMU (Fiumalbi et al., 2022).
2. *Middle-level control layer:* The middle-level controller uses the information measured by the onboard sensors and provided from the high-level control layer to (i) compute a continuous estimate of the gait phase, and (ii) send the reference motor commands to the low-level controller (Figure 5(b)).
  The continuous gait phase estimation is computed by a pool of Adaptive Oscillators (AOs) (Yan et al., 2017) with non-linear kernel-based filtering. The phase 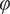 ranges from 0% to 100% within a stride, and it is reset at each FC. The AOs are synchronized with the rotation of the foot along the sagittal plane, estimated online through the Madgwick sensor fusion algorithm (Madgwick, 2010).
  The WRL TTP actuation is managed through a library of motor commands, determined and tuned during preliminary tests on healthy subjects. During the whole gait cycle, the middle-level controller provides as output (i) the low-level control mode ( 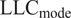 ), either *velocity control* or *zero current control*, and (ii) the respective desired low-level control reference ( 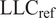 ). Notably, whenever the WRL TTP is controlled in *zero current*, the 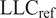 is set to 0 A.
3. *Low-level control layer*: When controlled in 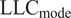 *velocity control*, the error between 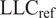 and the measured velocity of the motor is used as input for a proportional-derivative (PD) regulator, returning an electrical desired current 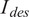 .

The WRL TTP can be controlled in two operating modes: passive mode (PM) and active mode (AM).

- Passive Mode

When controlled in PM, the actuator is driven at negative velocity during the swing phase to unload the energy stored in the ESAR foot compressed by the user, to emulate the behaviour of a passive device (Figure 5(b)):

1. from FC to FO, the motor is controlled in *zero current*, thus the actuator does not provide additional deformation of the foot;
2. at FO, the 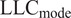 is set to *velocity control* and the 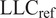 is instantaneously set to −2000 rpm to unload the residual elastic energy in swing;
3. the motor control mode is set to *zero current* whenever the foot is in a neutral, unloaded, position, which is detected when the camshaft angle is lower than a manually tuned threshold angle 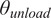 , close to the lower instability point 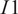 of the 4-bar linkage:

(2)

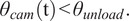

1. Active Mode

The AM control strategy drives the actuator during stance to bring the four-bar linkage over the upper instability point 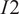 to return the energy during the push-off phase. The active loading is controlled by tuning a desired stride phase 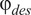 at which the energy should be returned (desired *flipover* phase). This control strategy ensures an adaptive energy release throughout the subject’s walk (Figure 5(b)):

1. from FC, the prosthesis is controlled in *zero current* until a set stride phase value is reached:

(3)

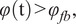

where 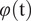 (%) is the online stride phase estimated by the AOs, and 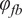 (%) is a feedback threshold used to command the engagement of the motor. The value of 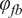 is initialized to 20% and updated at each stride according to the rule described in point 3 to make the prosthesis comply with different loading conditions;

1. when the equation in point 1 is met, the 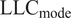 is set to *velocity control* with a 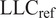 of 6000 rpm to run the motor at the maximum velocity, injecting energy in the elastic structure of the foot until the camshaft angle reaches 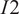 :

(4)

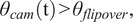

where 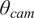 is the angle measured by the camshaft encoder, and the threshold angle 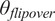 is the value of the camshaft angle when the linkage is at point 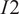 , manually set by the experimenters depending on the stroke length. At this time, the phase estimated by the AOs is stored as the actual flipover phase ( 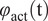 );

1. the control system computes the difference between 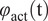 and the desired value 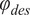 to update the feedback threshold 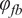 for the upcoming stride, according to the following rules:

(5)

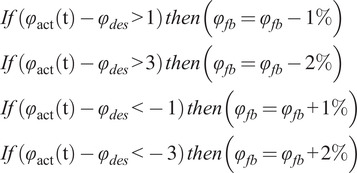



In particular, if 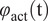 is greater than 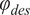 , 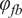 is lowered to engage the motor earlier during the next stride. Conversely, if 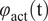 is lower than 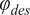 , 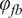 is increased to delay the motor engagement in the next stride.

1. the prosthesis is controlled in *zero current* from the flipover to FO, that is, the energy return phase. In this phase, the energy is rapidly returned thanks to the free motion of the overrunning clutch;
2. in the swing phase, the WRL TTP is controlled as in PM to reposition the foot in a neutral position.

## 3. Experiments and results

### 3.1. Concept verification with transtibial amputee subjects

**Figure 6.**
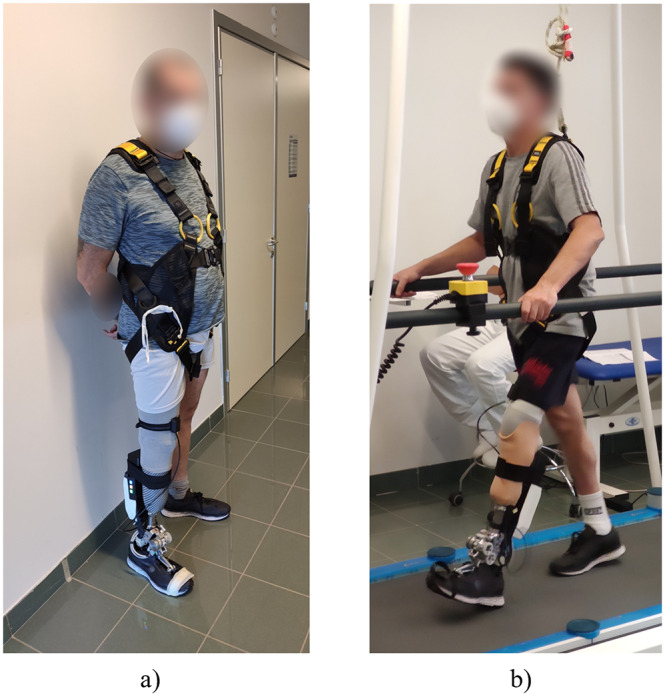
(a) Photograph of subject 1 wearing the WRL TTP. (b) Photograph of subject 2 using the WRL TTP during the verification tests.

Two transtibial amputees (Table 1) with a residual mobility level of K3/K4 (medicare functional classification levels (Gailey et al., 2002)) were recruited to perform treadmill walking to assess the ability of the WRL TTP to assist gait in different operating conditions (Figure 6). The experimental activities were approved by the Area Vasta Toscana Centro Ethics Committee (study number 16678), and conducted at IRCCS Fondazione Don Carlo Gnocchi (Florence, Italy). Tests were performed after an initial process of tuning and familiarization with the device. The WRL TTP was fixed and aligned on the socket of each subject by a prosthetist, and the thigh IMU was fastened above the knee along the sagittal plane of the leg using an elastic band. The stroke regulation was then adjusted after assessing the comfortable walking speed and the foot deformation produced by the user. The value of the stroke regulation was set to release the retained energy exclusively when controlling the prosthesis in AM: this ensured that the assistive action of the motor additionally deformed the foot, allowing the linkage to reach the flipover. Conversely, in PM the flipover was never reached, and the motor was driven backwards to unload the structure.

**Table 1.** Subjects’ general information and test parameters

ID	Sex	Age	Weight (kg)	Height (cm)	Year of amputation	Amputation side	Prescribed prosthesis	Self-selected speed (km/h)	Flipover phase (%)	Stroke regulation (mm)
1	M	52	83	182	2015	Right	Elan (Blatchford, UK)	3.5	45	11.2
2	M	45	63	172	2014	Left	Vari-Flex (Ossur, Iceland)	2.8	46	6.6

The tuning process of the flipover phase in AM ( 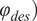 started from the average phase of the dorsiflexion peak (i.e., the beginning of push-off) in PM and was fine-tuned according to the user feedback. An *early* return phase 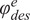 and a *late* return phase 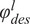 were also defined respectively as a decrement and an increment of 5% of the desired flipover phase 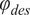 . The walking speeds for the test were selected following the procedure described by Nagano et al. (2013):

1. the subject started walking in AM at a comfortable speed;
2. the treadmill speed was increased by 0.3 km/h every ten strides until the subject had to stop due to excessive physical effort. The speed was then further increased by 0.3 km/h and noted as maximum speed;
3. the treadmill speed was brought to the initial value;
4. the treadmill speed was decreased by 0.3 km/h every ten strides until it became too uncomfortable to walk. The speed was then further reduced by 0.3 km/h and noted as minimum speed;
5. steps from 1 to 4 were repeated three times, and the self-selected speed ( 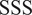 ) was set as the average value among the collected maxima and minima.

To demonstrate the capability of the prosthesis to continuously adapt to different speeds, once the 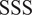 was set, the subject was asked to walk on the treadmill at three increasing speeds without taking breaks: the self-selected one, a speed 10% higher ( 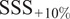 ), and one 20% higher ( 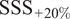 ). The subject walked at each speed for 60 s after the treadmill reached the steady-state speed. This procedure was repeated five times: in the first and the last trial, the prosthesis was operated in PM, whereas in the other trials, the operation mode of the prosthesis was AM, setting the three desired flipover phases 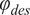 , 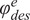 and 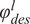 in a randomized order. The treadmill was equipped with the Optogait system (Microgate S.r.l., Italy) to extract relevant spatiotemporal gait parameters.

To have an insight into the end-users’ opinion on the prosthesis, at the end of the trial the subjects were asked to fill in an ad-hoc Visual Analog Scale (VAS) (Chiarotto et al., 2019) for three items (weight, comfort, and safety).

### 3.2. Data analysis

In data post-processing, single strides were extracted by segmenting the acquired data between two consecutive FC events and normalized over 100 samples. For each stride, the mean and standard deviation were computed for five metrics. To assess the capability of the prosthesis to adapt to different loading conditions and stride durations, we analyzed the actual flipover phase 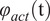 at the different commanded flipover phases and velocities. To evaluate the residual tension in the prosthesis, we measured the peak force measured by the load cell, at FO before commanding the active unload of the leaf spring of the ESAR foot. To evaluate the capability of the prosthesis to deform the structure additionally and inject energy, we estimated the peak dorsiflexion ankle angle and the energy injected by the actuator (computed as the difference between the work exchange during AM and PM strides estimated offline by exploiting the model presented in the Supplementary Material). To preliminary assess behavioral differences between the two operating modalities, we computed the cadence of the subjects, as used in other clinical assessments and gait training (TIRR SCI Clinical Exoskeleton Group et al., 2018; Bunge et al., 2021). Moreover, we computed the temporal and spatial symmetry indexes (SI) as
(6)

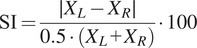

where 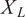 and 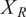 were either the stride length (spatial SI) or the stride time (temporal SI) of the left and right limb, respectively, extracted from the Optogait system (Błażkiewicz et al., 2014). With this definition, a SI of 0% indicates perfect symmetry.

For results visualization, a representative stride (one for each control modality and speed) was chosen as the one with lower RMS error with respect to the median profile computed from all the strides extracted.

### 3.3. Results

**Figure 7.**
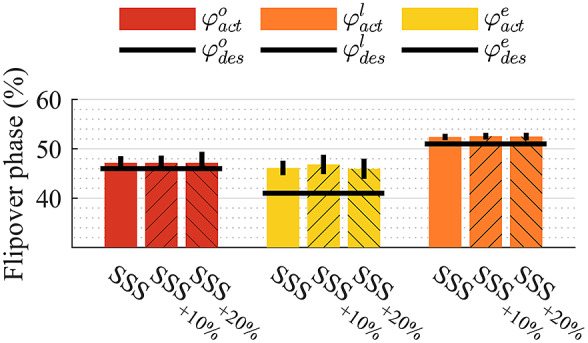
The flipover phase’s mean and standard deviation across the different walking speeds and desired commanded phases.

Figure 7 shows the mean and standard deviation of the actual flipover phase 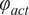 , for both subjects, at different velocities and for different values of desired flipover phase, displayed by the black horizontal lines. For the desired flipover phase 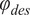 , at the 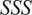 (see Table 1), the actual flipover phase was 46.63 ± 0.88% for subject 1 and 47.17 ± 1.36% for subject 2, respectively.

**Figure 8.**
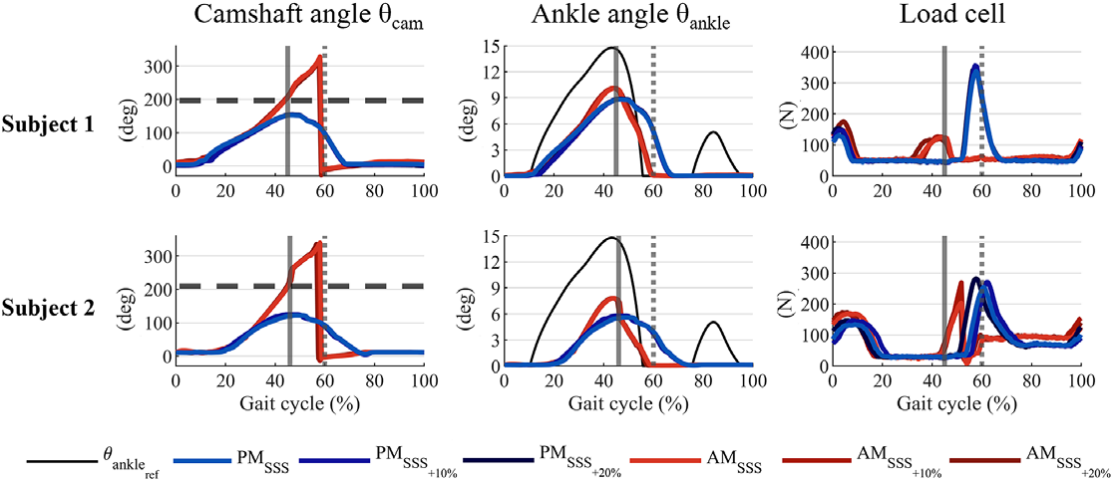
Camshaft angle ( 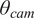 ), ankle joint angle ( 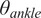 ), and load cell profiles for representative strides in PM and AM at different speeds. The solid grey vertical line indicates the desired flipover phase, the beginning of the energy return. The grey dotted vertical line indicates the FO event. The horizontal dashed line represents the position of point I2, that is, the reaching of the flipover.

In Figure 8, the profiles of the camshaft encoder angle, the estimated ankle angle, and the load cell are shown for representative strides at the flipover phase 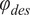 , for each treadmill speed. When the prosthesis was controlled in PM, the average peak in the load cell signal at FO was 318 ± 24 N for subject 1, and 238 ± 36 N for subject 2. When controlling the prosthesis in AM, the load cell peaks at FO lowered, reaching an average value of 85 ± 49 N and 91 ± 30 for subjects 1 and 2, respectively.

The estimated ankle angle for PM and AM is also compared with the reference data of joint angle for a healthy ankle extracted from Bovi et al. (2011), restricted to dorsiflexion ( 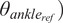 . When controlled in AM, the prosthesis increased the average peak of the estimated joint angle from 8.66 ± 0.27 deg to 10.02 ± 0.37 deg for subject 1 and from 5.58 ± 0.31 deg to 7.65 ± 0.68 deg for subject 2.

**Figure 9.**
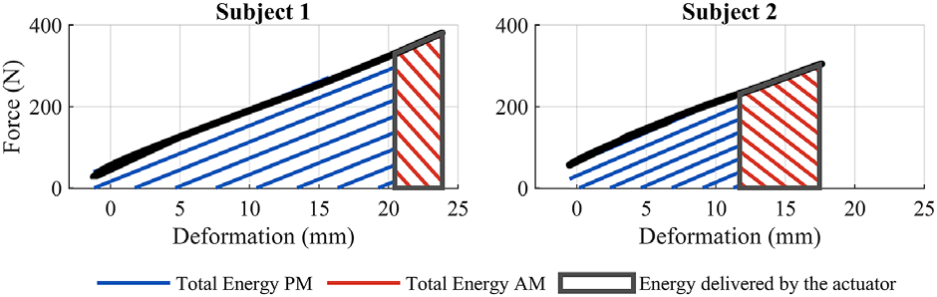
Averaged profile of the relation between the displacement 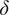 and the force 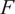 . The area underneath the profile is the energy exerted on the prosthetic foot. The blue area is the energy stored in the device in PM, the red area is the energy stored in the device in AM. The difference between the two areas is the energy injected by the actuator.

The estimated mean positive energy of the ankle in PM and AM (Figure 9) was respectively 3.85 ± 0.04 J and 5.23 ± 0.05 J for subject 1, and 1.74 ± 0.05 J and 3.34 ± 0.03 J for subject 2. The energy injected by the actuator was approximately 1.38 J for subject 1 and 1.6 J for subject 2.

**Figure 10.**
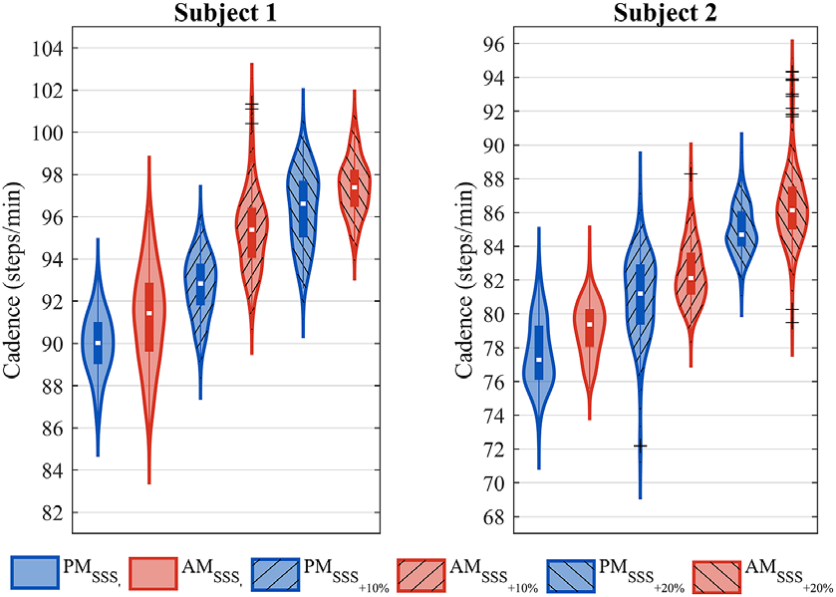
Violin plot of the instantaneous cadence in PM and AM, for all the treadmill velocities tested.

Figure 10 shows the distribution of the instantaneous cadence for the desired flipover phase 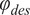 at the three different velocities. Blue violin plots are used for PM operating mode, while red ones indicate AM operating mode. The cadence increased on average of about 2% when using the prosthesis in AM with respect to PM, for each subject.

**Figure 11.**
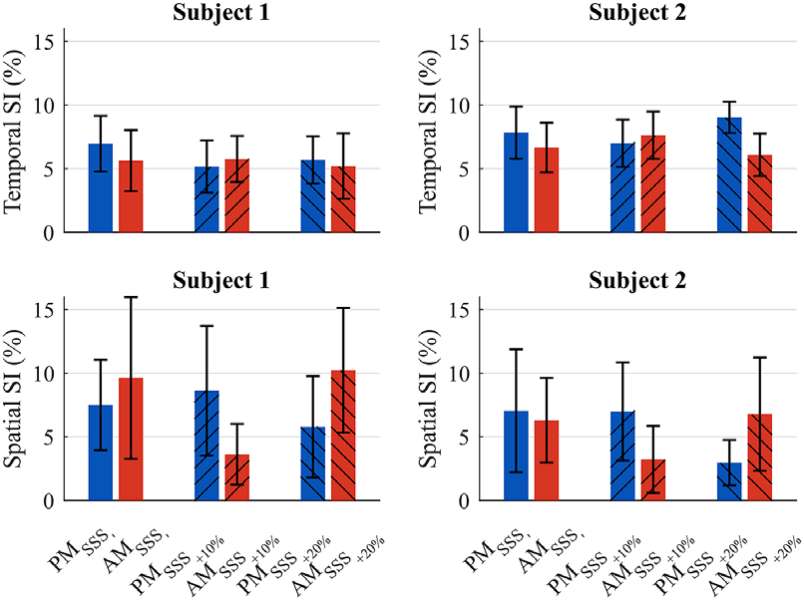
Mean temporal and spatial SI for both subjects in PM and AM, for all the treadmill velocities tested.

Figure 11 shows the temporal SI and the spatial SI for the desired flipover phase 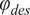 at the three different velocities. Blue bars represent the SI during PM operating mode, while red bars indicate AM operating mode. All the mean SI were below 11%, with few differences among the tested conditions.

## 4. Discussion

This study presents a low-power prosthetic module designed to enhance the energy storage and return capabilities of an ESAR passive foot. Rather than relying on additional elastic elements to accumulate energy (Collins and Kuo, 2010; Cherelle et al., 2017), the developed parallel elastic actuation paradigm exploits the elastic nature of the ESAR foot structure. This approach allows the concept of the WRL TTP to (i) always ensure the safety and essential mobility of the standard ESAR foot, even in case of malfunction or shutdown, and (ii) adapt the module to fit different passive feet structures.

### 4.1. Verification of the proposed device

From the preliminary verification with two amputee subjects, we proved the ability of the prosthesis to automatically adapt to changes in the stride duration and so in the flipover phase, and different user’s loading conditions, as it happens with different walking speeds (Figure 7). When controlling the prosthesis in AM, the camshaft angle always overcame the position of point I2 (Figure 8). The flipover event was reached at each stride with a repeatable behaviour.

The main limitation in the adaptation capability resides in the physical limits given by (i) the motor power and (ii) the net time of loading operation of the motor. These aspects ultimately define the maximum energy the motor can inject within each stride. Indeed, the actuation can compress the ESAR foot only in the time window between the foot flat and the flipover phase. As a consequence, the minimum energy return phase that the prosthesis can physically reach is constrained by (i) the amount of energy exerted solely by the user on the carbon foot, (ii) the position of the upper instability point 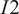 , defined by the stroke regulation, and (iii) the amount of energy that the motor can inject within the stance phase. For instance, a stroke regulation that aims at anticipating the phase of the energy return (i.e., a stroke shifted towards the minimum value 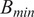 ) relies more on the loading action provided by the user, and therefore less energy is needed by the motor to reach the flipover position. Regardless, with the chosen motor, the active contribution of the device cannot be used for an energy return earlier than 40% of the stride, even when the motor is immediately engaged at FC.

The injection of additional energy can be seen in Figure 8: the tension on the foot measured by the load cell data in AM during mid-stance reflects the loading action of the actuator. Moreover, the release of energy was verified by the decrease of the peak in the load cell signal at FO when the WRL TTP was controlled in AM. The lower peak indicates less load in the prosthesis, demonstrating the effective transfer of energy to the subject. On the contrary, in PM trials, the energy stored in the foot is mechanically held until FO. In this case, the tension measured on the foot structure increases as the user’s load fades during push-off, measuring a peak in the tension on the foot’s structure at FO. The PM control strategy exploits the controlled unloading of the WRL TTP to improve the toe clearance during the early swing at the cost of a partial dissipation of the push-off energy. As shown in Figure 8, the ankle angle remains dorsiflexed up to mid-swing (70–80% of the gait phase).

We also verified the device’s capability to actively inject energy into the gait by estimating the energy injected by the actuator from the offline implemented model. The estimated energy stored in the foot increased when controlling the WRL TTP in AM. We found an increase in the energy stored of 26% for subject 1 and 45% for subject 2 compared to the one exerted by the sole action of the amputee during PM trials. Moreover, the energy injected by the actuation unit has shown to be approximately the 5% of the total push-off work of a healthy subject (Collins and Kuo, 2010).

We assessed the ability of the device to additionally deform the structure through the estimate of the ankle angle. The compression induced in the structure of the ESAR foot in the AM trials resulted in an increase in the dorsiflexion peak by 15.2% for subject 1 and 41.6% for subject 2. Despite proving to increase the natural dorsiflexion of the ESAR foot, the geometry of the linkage denies any plantarflexion in the passive foot. When walking with the sole ESAR foot, the tensile forces acting on the foot’s structure result in a plantarflexion during the initial phases of the gait cycle, namely from the foot contact to the foot flat (Childers and Takahashi, 2018; Tomkin et al., 2018). Nevertheless, the transmission of the WRL TTP is designed to bind the minimum position of the forefoot at the lower instability point of the four-bar linkage 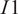 , corresponding to the neutral position of the foot. As a consequence, the tensile action performed by the user until the foot flat is translated into tension on the structure of the WRL TTP forefoot, as measured by the load cell (Figure 8), while the heel portion of the ESAR foot keeps its damping capability at the heel strike.

As shown in Figure 10, both subjects showed an increase in cadence when walking in AM with respect to PM, confirming that the prosthesis injected energy into the gait of both users. Given the small sample size and the high level of mobility of the enrolled subjects (K3/K4), no trends were visible in the SI, as shown in Figure 11. Moreover, the values were within normative ranges of healthy subjects (Błażkiewicz et al., 2014).

### 4.2. Limitations and future work

Although the experimental results proved the concept of the actuation of the WRL TTP, further studies are necessary to assess the impact of the assistance provided by the prosthetic module on the gait of a broader population. Such trials could focus on evaluating the efficacy of the WRL TTP on ecological test conditions by measuring the metabolic cost of walking, the biomechanical effects of the assistance on the residual and contralateral limb, as well as the effects on symmetry, step length, and stride duration. These trials will be useful to determine whether the device’s assistance can provide beneficial effects over off-the-shelf ESAR feet, at the expense of the added weight. Moreover, it would be interesting to enroll low-mobility amputees. In fact, the presented device could also be beneficial for K1/K2 level amputees since the literature suggests that amputees with a low level of mobility may not benefit from the energy return potential of elastic feet because of the noncontrolled timing and magnitude of the energy return (Segal et al., 2012).

In completing the VAS, both subjects reported no issues in perceived comfort and security but rated the prosthesis as heavy. Indeed, the overall weight of 2745 g for the first WRL TTP prototype is a major limiting factor on the potential benefits of the assistance on the user’s gait (Browning et al., 2007). The presented prosthetic module adds 2080 g to the structure of the standard ESAR foot, resulting in overall mass distribution and an encumbrance that is closer to one of fully active ankle-foot prostheses than to one of low-power, semi-active devices (Au et al., 2009; Collins and Kuo, 2010; Gabert et al., 2020). However, the weight of the sole mechanical assembly is 630 g, which is in line with the one reported by other semi-active devices in the state-of-the-art (Lenzi et al., 2019) or by commercial devices (Proprio Foot, Ossür®, Reykjavik, Iceland) (Meridium, Ottobock®, Duderstadt, Germany). Further optimization iterations are required to reduce the weight due to the sensors, the control electronics, and increase the duration of the battery. Nonetheless, the underconstrained differential mechanism enables the use of the prosthesis in daily life scenarios, allowing the user to walk when the actuator is turned off.

The current prototype also shows room for improvement in the design of the transmission. The design of the four-bar linkage and the differential system on the forefoot resulted in an increased instep for the foot, which could not fit properly in a regular shoe. Therefore, a future prototype might focus on designing a linkage closer to the bottom plate of the ESAR foot, decreasing the total encumbrance of the prosthesis. Alternatively, it would be possible to move the mechanism on the side of the foot, placing the motor near the leaf spring and developing a different linkage to connect the motor to the tip of the foot.

Moreover, the WRL TTP might be suitable to assist different locomotion activities of daily living, such as upslope walking or stair ascent. Differently from walking activities, during stair ascent, the motor of the WRL TTP might be engaged in the late swing. Indeed, given the stiffening of the structure of the ESAR foot due to the compression, such loading action would allow a stiffer foot at FC, possibly resulting in an increased sense of stability for the amputee and improved proprioception of the ground. Notably, an online classification algorithm for locomotion mode recognition might be, in this case, required to allow an automatic switch of the control strategy.

## 5. Conclusion

This article described a novel concept of a low-power active prosthetic module that exploits the elastic structure of an ESAR foot to implement a parallel elastic actuation. The ankle-foot prosthesis can operate as a bare passive foot, with the option to actively inject energy into the foot during stance to return the total energy during the push-off and improve forward propulsion. Preliminary verification of the proposed prototype on two transtibial amputees showed an average increase of the energy return of 35.5% in AM with respect to PM. The control algorithm implemented adapts to the user’s gait pattern and loading conditions to provide additional energy at the desired stride phase. Future work will improve the current prototype by reducing its size and weight and updating the control strategy to include different locomotion modes. Notably, an implementation of machine learning techniques could achieve an automatic identification of transitions between locomotion modes. Finally, we plan to perform a validation of the device to quantify clinical outcomes and end-user preferences.

## Data Availability

The datasets generated during and/or analyzed during the current study are available from the corresponding author on reasonable request.

## Supplementary material

For supplementary material accompanying this paper visit https://doi.org/10.1017/wtc.2023.13. click here to view supplementary material

## References

[r1] Au SK, Weber J and Herr H (2009) Powered ankle-foot prosthesis improves walking metabolic economy. IEEE Transactions on Robotics 25(1), 51–66. 10.1109/TRO.2008.2008747

[r2] Błażkiewicz M, Wiszomirska I and Wit A (2014) Comparison of four methods of calculating the symmetry of spatial-temporal parameters of gait [PDF]. Acta of Bioengineering and Biomechanics 16, 29–35. 10.5277/ABB140104 24708092

[r3] Bovi G, Rabuffetti M, Mazzoleni P and Ferrarin M (2011) A multiple-task gait analysis approach: Kinematic, kinetic and EMG reference data for healthy young and adult subjects. Gait and Posture 33(1), 6–13. 10.1016/j.gaitpost.2010.08.009 21123071

[r4] Browning RC, Modica JR, Kram R and Goswami A (2007) The effects of adding mass to the legs on the energetics and biomechanics of walking. Medicine and Science in Sports and Exercise 39(3), 515–525. 10.1249/mss.0b013e31802b3562 17473778

[r5] Bunge LR, Davidson AJ, Helmore BR, Mavrandonis AD, Page TD, Schuster-Bayly TR and Kumar S (2021) Effectiveness of powered exoskeleton use on gait in individuals with cerebral palsy: A systematic review. PLoS One 16, 1–24. 10.1371/journal.pone.0252193 PMC8153467 34038471

[r6] Caputo JM and Collins SH (2014) Prosthetic ankle push-off work reduces metabolic rate but not collision work in non-amputee walking. Scientific Reports 4, 37–41. 10.1038/srep07213 PMC4252906 25467389

[r7] Cherelle P, Grosu V, Flynn L, Junius K, Moltedo M, Vanderborght B and Lefeber D (2017) The ankle mimicking prosthetic foot 3—Locking mechanisms, actuator design, control and experiments with an amputee. Robotics and Autonomous Systems 91, 327–336. 10.1016/j.robot.2017.02.004

[r8] Chiarotto A, Maxwell LJ, Ostelo RW, Boers M, Tugwell P and Terwee CB (2019) Measurement properties of visual analogue scale, numeric rating scale, and pain severity subscale of the brief pain inventory in patients with low back pain: A systematic review. The Journal of Pain 20(3), 245–263. 10.1016/j.jpain.2018.07.009 30099210

[r9] Childers WL and Takahashi KZ (2018) Increasing prosthetic foot energy return affects whole-body mechanics during walking on level ground and slopes. Scientific Reports 8(1), 1–12. 10.1038/s41598-018-23705-8 29599517 PMC5876366

[r10] Collins SH and Kuo AD (2005) Controlled energy storage and return prostheses reduces metabolic cost of walking. In ISB XXth Congress - ASB 29th Annual Meeting, p. 804.

[r11] Collins SH and Kuo AD (2010) Recycling energy to restore impaired ankle function during human walking. PLoS One 5(2), e9307. 10.1371/journal.pone.0009307 20174659 PMC2822861

[r12] Fantozzi M, Baldoni A, Crea S and Vitiello N (2018) Wearable robotic device for moving a user.

[r13] Ferris AE, Aldridge JM, Rábago CA and Wilken JM (2012) Evaluation of a powered ankle-foot prosthetic system during walking. Archives of Physical Medicine and Rehabilitation 93(11), 1911–1918. 10.1016/j.apmr.2012.06.009 22732369

[r14] Fiumalbi T, Martini E, Papapicco V, Agnello FD, Mazzarini A, Baldoni A, Gruppioni E, Crea S and Vitiello N (2022) A multimodal sensory apparatus for robotic prosthetic feet combining optoelectronic pressure transducers and IMU. Sensors (Basel) 22, 1731. 35270877 10.3390/s22051731 PMC8914932

[r15] Gabert L, Hood S, Tran M, Cempini M and Lenzi T (2020) A compact, lightweight robotic ankle-foot prosthesis: Featuring a powered polycentric design. IEEE Robotics and Automation Magazine 27(1), 87–102. 10.1109/MRA.2019.2955740 33790527 PMC8009500

[r16] Gailey RS, Roach KE, Applegate EB, Cho B, Cunniffe B, Licht S, Maguire M and Nash MS (2002) The amputee mobility predictor: An instrument to assess determinants of the lower-limb amputee’s ability to ambulate. Archives of Physical Medicine and Rehabilitation 83(5), 613–627. 10.1053/apmr.2002.32309 11994800

[r17] Glanzer EM and Adamczyk PG (2018) Design and validation of a semi-active variable stiffness foot prosthesis. IEEE Transactions on Neural Systems and Rehabilitation Engineering 26(12), 2351–2359. 10.1109/TNSRE.2018.2877962 30371376 PMC6289620

[r18] Hitt JK, Sugar TG, Holgate M and Bellman R (2010) An active foot-ankle prosthesis with biomechanical energy regeneration. Journal of Medical Devices, Transactions of the ASME 4(1), 1–9. 10.1115/1.4001139

[r19] Lenzi T, Cempini M, Hargrove LJ and Kuiken TA (2019) Design, development, and validation of a lightweight Nonbackdrivable robotic ankle prosthesis. IEEE/ASME Transactions on Mechatronics 24(2), 471–482. 10.1109/TMECH.2019.2892609

[r20] Lenzi T, Cempini M, Newkirk J, Hargrove LJ and Kuiken TA (2017) A lightweight robotic ankle prosthesis with non-backdrivable cam-based transmission. IEEE International Conference on Rehabilitation Robotics 2017, 1142–1147. 10.1109/ICORR.2017.8009403 28813975

[r21] Lu S, Zlatanov D, Ding X and Molfino R (2014) A new family of deployable mechanisms based on the Hoekens linkage. Mechanism and Machine Theory 73, 130–153. 10.1016/j.mechmachtheory.2013.10.007

[r22] Madgwick SOH (2010) An efficient orientation filter for inertial and inertial/magnetic sensor arrays. Report x-io and University of Bristol (UK) 25, 113–118.

[r23] Nagano H, Begg RK, Sparrow WA and Taylor S (2013) A comparison of treadmill and overground walking effects on step cycle asymmetry in young and older individuals. Journal of Applied Biomechanics 29(2), 188–193. 10.1123/jab.29.2.188 22814355

[r24] Narres M, Kvitkina T, Claessen H, Droste S, Schuster B, Morbach S, Rümenapf G, Van Acker K and Icks A (2017) Incidence of lower extremity amputations in the diabetic compared with the non-diabetic population: A systematic review. PLoS One 12(8). 10.1371/journal.pone.0182081 PMC5573217 28846690

[r25] Sawers A and Hahn ME (2011) Trajectory of the center of rotation in non-articulated energy storage and return prosthetic feet. Journal of Biomechanics 44(9), 1673–1677. 10.1016/j.jbiomech.2011.03.028 21481878

[r26] Segal AD, Zelik KE, Klute GK, Morgenroth DC, Hahn ME, Orendurff MS, et al. (2012) The effects of a controlled energy storage and return prototype prosthetic foot on transtibial amputee ambulation. Human Movement Science 31(4), 918–931. 10.1016/j.humov.2011.08.005 22100728 PMC4302415

[r27] Shepherd MK and Rouse EJ (2017) The VSPA foot: A quasi-passive ankle-foot prosthesis with continuously variable stiffness. IEEE Transactions on Neural Systems and Rehabilitation Engineering 25(12), 2375–2386. 10.1109/TNSRE.2017.2750113 28885156

[r28] TIRR SCI Clinical Exoskeleton Group, Chang S-H, Afzal T, Berliner J and Francisco GE (2018) Exoskeleton-assisted gait training to improve gait in individuals with spinal cord injury: A pilot randomized study. Pilot and Feasibility Studies 4(1), 62. 10.1186/s40814-018-0247-y 29556414 PMC5839068

[r29] Tomkin M, Gholizadeh H, Sinitski E and Lemaire ED (2018) Transtibial amputee gait with the pro-flex foot during level, decline, and incline walking. Canadian Prosthetics & Orthotics Journal 1(2), 1–2. 10.33137/cpoj.v1i2.32003

[r30] Tucker MR, Olivier J, Pagel A, Bleuler H, Bouri M, Lambercy O, et al. (2015) Control strategies for active lower extremity prosthetics and orthotics: A review. Journal of Neuroengineering and Rehabilitation 12(1), 1. 10.1186/1743-0003-12-1 25557982 PMC4326520

[r31] Waters RL and Mulroy S (1999) The energy expenditure of normal and pathologic gait. Gait and Posture 9(3), 207–231. 10.1016/S0966-6362(99)00009-0 10575082

[r32] Whittle MW (2014) Gait Analysis: An Introduction. Oxford: Butterworth-Heinemann.

[r33] Windrich M, Grimmer M, Christ O, Rinderknecht S and Beckerle P (2016) Active lower limb prosthetics: A systematic review of design issues and solutions. Biomedical Engineering Online 15(3), 5–19. 10.1186/s12938-016-0284-9 28105948 PMC5249019

[r34] Yan T, Parri A, Ruiz Garate V, Cempini M, Ronsse R and Vitiello N (2017) An oscillator-based smooth real-time estimate of gait phase for wearable robotics. Autonomous Robots 41(3), 759–774. 10.1007/s10514-016-9566-0

[r35] Ziegler-Graham K, MacKenzie EJ, Ephraim PL, Travison TG and Brookmeyer R (2008) Estimating the prevalence of limb loss in the United States: 2005 to 2050. Archives of Physical Medicine and Rehabilitation 89(3), 422–429. 10.1016/j.apmr.2007.11.005 18295618

