# Supplementary material for: A low-power ankle-foot prosthesis for push-off enhancement
Source: Wearable Technol. 2023 Jun 15;4:e18. doi: 10.1017/wtc.2023.13 (PMC10936261; doi:10.1017/wtc.2023.13)
Supplement: Supplementary file 1 [file S2631717623000130sup001.docx]

# Supplementary material

In this appendix, details about the WRL TTP kinematic and kinetic modelling are presented.

## Kinematic model


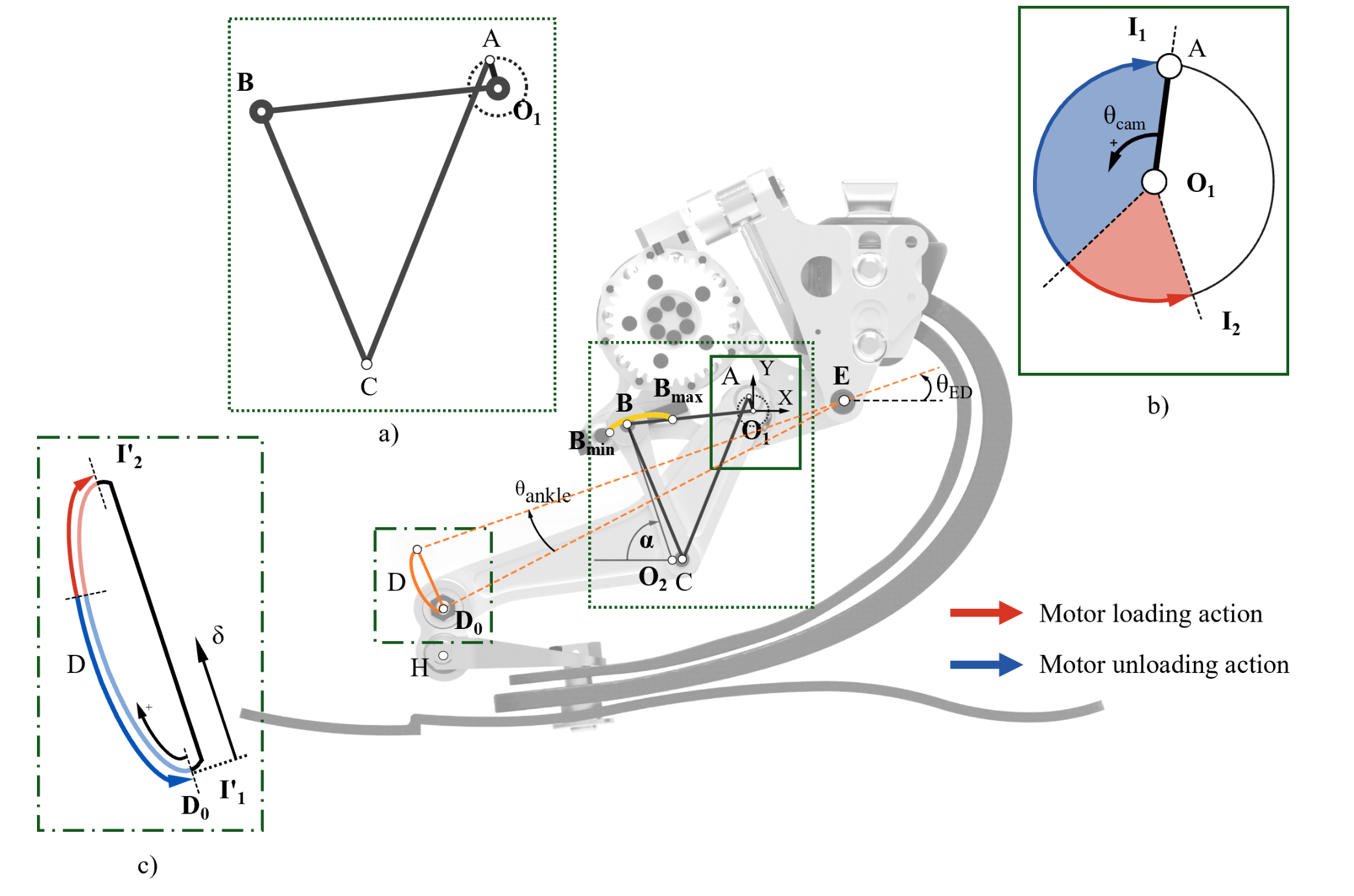


Figure A1. Mechanical assembly of the WRL TTP. a) Segments of the four-bar linkage O_1_-A-C-B. b) Camshaft trajectory with the two instability points of the mechanism I1 and I2. c) End-effector trajectory. Bold letters correspond to fixed points.

A kinematic model was designed to compute an estimate of the ankle joint angle $\theta_{\mathrm{ankle}}$ (Figure A1) and the displacement $\delta$ of the end-effector $D$ from its lowest position $D_{0}$. The model requires as input (i) the camshaft angle $\theta_{cam}$, (ii) the stroke regulation $BB_{min}$_,_ and (iii) the geometrical parameters defining the characteristic kinematic chain:

$\left\{ \begin{aligned} O_{1}A=4.6 mm \\ AC=50 mm \\ BC=42 mm \\ CD=70.54 mm \\ AD=107.02 mm \\ BO_{2}=41.72 mm \end{aligned} \right..$ ($SEQ Equation \backslash* ARABIC$ $1$)

The point $O_{2}$ is the center of the arc in which $B$ can be moved to set the stroke regulation.

The ankle joint angle $\theta_{\mathrm{ankle}}$ and the displacement $\delta$ are computed following the steps described below:

1. the angle $\alpha$, defined between the segment $BO_{2}$ and the X-axis, is computed given the stroke regulation $BB_{min}$:

$\alpha= \begin{aligned} \mathrm{asin} \left( \frac{\frac{1}{2}{BB}_{\min}}{BO_{2}} \right)+\alpha_{min} \end{aligned}$. ($SEQ Equation \backslash* ARABIC$ $2$)

Where $\alpha_{min}$is equal to 72.4° and it is the value of $\alpha$ when the stroke regulation is minimum (${BB}_{\min}=0$). Depending on the stroke regulation, $\alpha$ can vary between $\alpha_{min}$ and 89.8 degrees.

1. The coordinates of the points $A$ and $B$ of the four-bar linkage are computed as a function of $\alpha,$ and the camshaft angle $\theta_{cam}$:

$\left\{ \begin{aligned} X_{A}=O_{1}A\cdot\cos\left( \theta_{cam} \right) \\ Y_{A}=O_{1}A\cdot\sin\left( \theta_{cam} \right) \\ X_{B}=X_{O2}-BO_{2}\cdot cos(\alpha) \\ Y_{B}=Y_{O2}+BO_{2}\cdot sin(\alpha) \end{aligned} \right.$. ($SEQ Equation \backslash* ARABIC$ $3$)

1. The coordinates of the point $C$ of the four-bar linkage are computed as the intersection between the circumference centered in $A$ with radius $AC$, and the circumference centered in $B$ with radius $BC$. Among the two possible intersections between the circumferences, the one with a lower Y-coordinate is chosen because it is the only one with physical meaning.
2. The coordinates of the end-effector $D$ are computed as the intersection between the circumference centered in $A$ with radius $AD$, and the one centered in $C$ with radius $CD$.
3. The angle $\theta_{\mathrm{ED}}$, i.e., the angle between the segment $ED$ and the X-axis, is computed as:

$\theta_{\mathrm{ED}}= \tan^{-1} \left( \frac{Y_{D}-Y_{E}}{X_{D}-X_{E}} \right)$. (4)

The coordinates ($X_{E}, Y_{E}$) of the fixed point $E$ are known since it is the center of rotation of the structure.

1. The estimate of the ankle angle $\theta_{\mathrm{ankle}}$can be found as:

$\theta_{\mathrm{ankle}}=\left| \theta_{\mathrm{ED}}-\theta_{\mathrm{EDmax}} \right|$, (5)

where $\theta_{\mathrm{EDmax}}$ is the angle $\theta_{\mathrm{ED}}$ when $D$ is at its lowest position ($D_{0}$). It is worth noting that, given the device's geometric constraints, the model can only estimate dorsiflexion angles up to 16 deg, depending on the specific stroke regulation set.

1. The displacement $\delta$ is computed as the distance between the point $D$ and its lowest position $D_{0}$:

$\left. \delta= \left( \frac{Y_{D}-(m_{0}\cdot X_{D}+q_{0})}{\sqrt{(1+m_{0}^{2})}} \right) \right.$, (6)

where $m_{0}=tan(\theta_{\mathrm{EDmax}})$ and $q_{0}=(Y_{E}-m_{tg}\cdot X_{E})$.

The plots in Figure A2 show the relationship between the camshaft angle $\theta_{cam}$and the model's outputs, for five different stroke regulations spanning the whole range of $\alpha$.


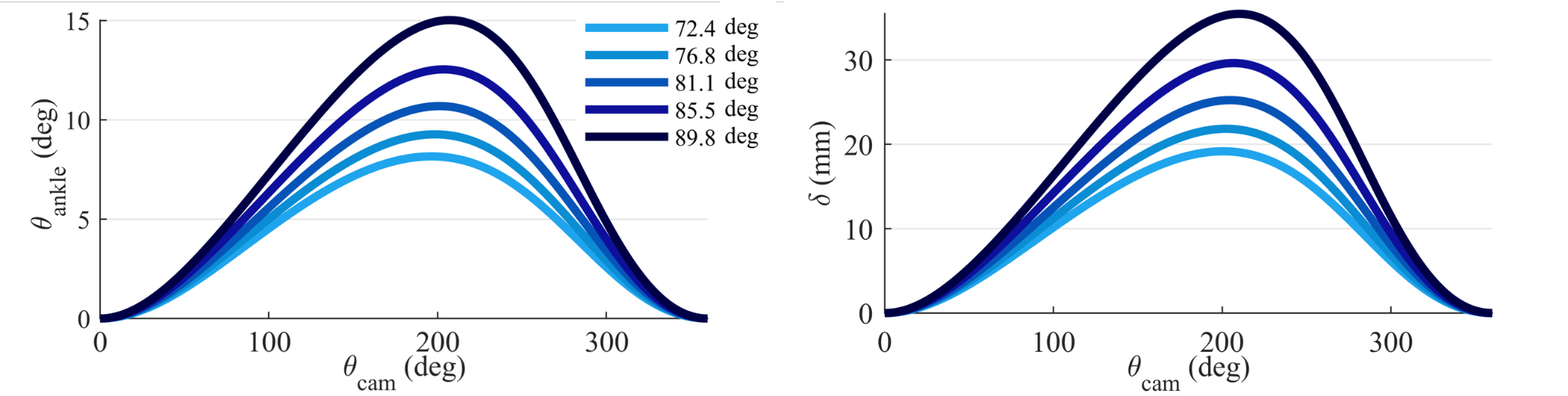


Figure A2. Ankle dorsiflexion angle $\theta_{ankle}$ and segment $\delta$ estimate as a function of the camshaft angle $\theta_{cam}$, for five different stroke regulations spanning the range of the parameter $\alpha$.

## Kinetic model

A kinetic model was designed to estimate the energy stored in the prosthesis. The energy is defined as the product of the force acting on the rigid link $HD$, which connects the ESAR foot to the actuation unit, and the displacement $\delta$:

$E=F_{\mathrm{HD}}\cdot\delta$. ($SEQ Equation \backslash* ARABIC$ $7$)

The link $HD$ is the segment in which the forces whose resultant is $F_{HD}$ are exchanged between the actuator and the ESAR foot. The $F_{HD}$ value was estimated from experimental results of uniaxial tests (INSTRON® 5960 Dual Column Table Frame), as a function of $\beta_{setup}$. The setup used to measure the forces and displacement of the prosthetic foot was conceived to lock the prosthesis between two parallel plates, connected by a parallelogram ($GG'F'F$) which keeps the upper plate horizontal along a trajectory $\xi$ when a downward motion is applied (Figure A3). The prosthesis was fixed on the setup with an initial angle $\gamma$ of 15 deg between the horizontal plate and the prosthesis ground plane (Figure A3, c), according to standard test conditions for commercial prostheses (International Organization for Standardization [ISO], 10328:2016). From the data collected during the uniaxial tests, $F_{HD}$ is computed implementing the following steps:

1. The horizontal reaction forces $R_{X}$ on the contact point $P$ is computed as a function of $\beta_{setup}$ (i.e., the angle of rotation of the links $GF$ and $G'F'$ with respect to the horizontal) and the vertical force $F_{V}$ applied by the uniaxial testbed.
2. By assuming rolling without sliding between the prosthetic foot and the upper plate of the setup, the vertical reaction force $R_{Y}$ on the contact point $P$ is computed as:

$R_{Y}=\frac{R_{X}}{f}$ (8)

where $f=0.19$ is the friction coefficient between rubber and aluminum.

1. The bending torque $T$ acting on the fixed point $I$ is found by combining the reaction forces $R_{X}$ and $R_{Y}$ as:

$T=R_{X}\cdot b_{X}+ R_{Y}\cdot b_{Y}$, (9)

where $b_{X}$ and $b_{Y}$ are the lever arms between $I$ and $R_{X}$ and $R_{Y}$, respectively. The lever arms change for any vertical displacement $\Delta y$ forced by the uniaxial testbed.

1. The lever arm $a$ is the distance between $I$ and the line passing through the link $HD$. Given the coordinates of 𝐷 from the kinematic model and the length of the link 𝐻𝐷 (13 mm) it is possible to geometrically retrieve the coordinates of point $H$ when a load is applied, thus the value of the lever arm $a$.
2. Lastly, the resultant force $F_{HD}$ along the link $HD$ is computed as:

$F_{HD}=\frac{T}{a}$. (10)

Therefore, for each vertical load ($F_{V}$) and vertical displacement ($\Delta y$) exerted by the testing machine, the energy $E$was computed.


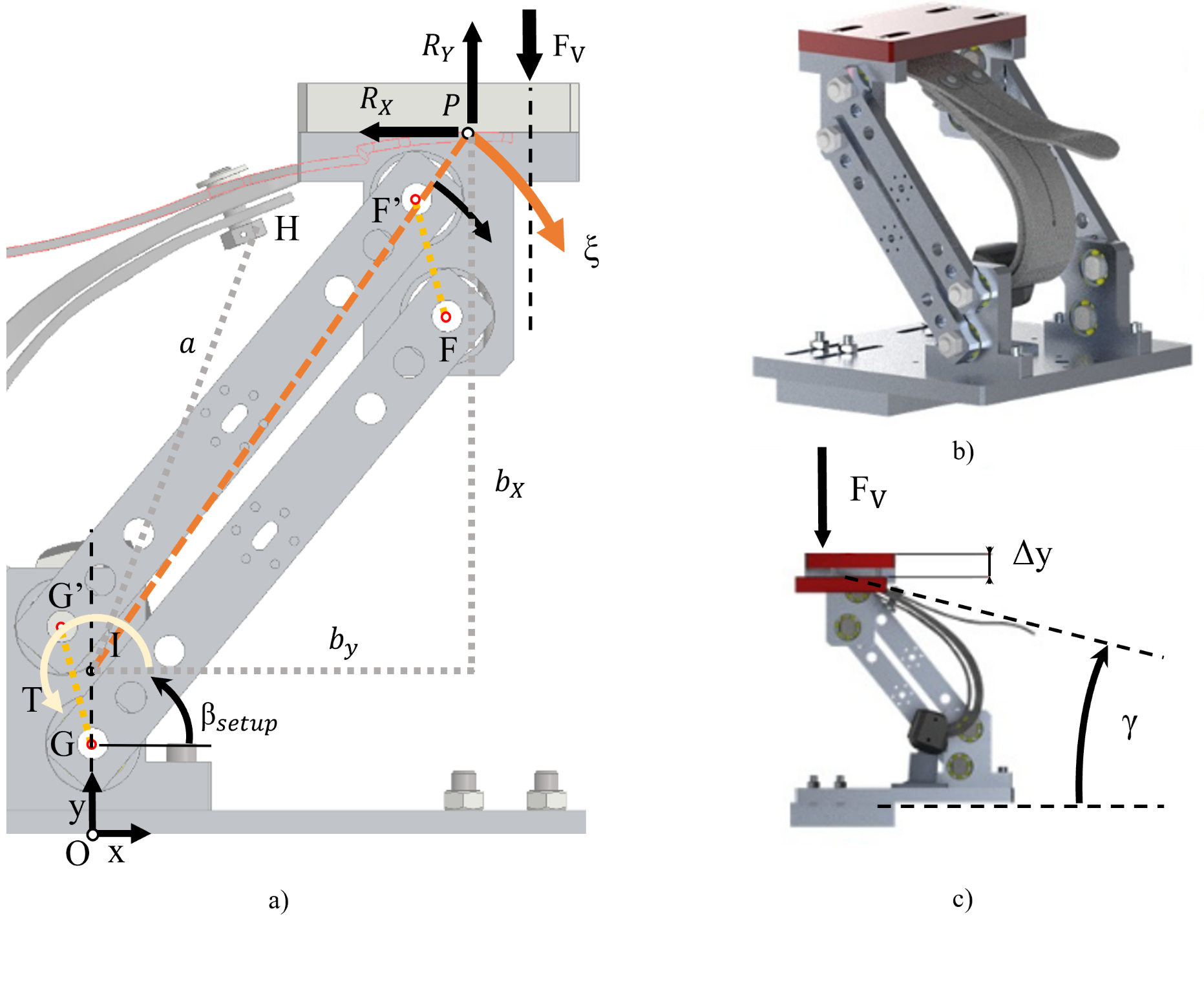


Figure A3. a) Setup with the main elements of the kinetic model. b) Rendering of the foot positioned inside the setup. c) Lateral view of the setup positioned on the testbed.

Since the actuation unit is parallel to the ESAR foot, it is not possible to directly quantify the actuator's net contribution in the total energy. Nevertheless, an offline estimate of the energy injected by the actuator in a gait cycle can be computed by merging the outputs of the kinematic and kinetic models, considering the following hypotheses:

- H1: the phase interval in which the amputee injects energy is limited between the foot contact (FC) and the phase at which the dorsiflexion peak occurs when the motor is not engaged. This corresponds to a camshaft movement across the blue region in Figure A1, b.
- H2: the contribution of the actuator is considered only after the loading action of the amputee. This corresponds to a camshaft movement across the red region in Figure A1, b.
- H3: the amputee loading action is constant among different strides.

Therefore, the work performed by the actuator can be defined as the difference between the energy injected into the prosthesis within a stride when the motor is engaged (the work exerted by the action of both the amputee and the actuator) and when it is not (the work exerted solely by the amputee).
